# Medical Societies

**Published:** 1877-08-20

**Authors:** 


MEDICAL SOCIETIES.—We have before adverted to the importance of Medical Societies. Almost every village—certainly every county—could establish a weekly, a monthly, or a semi-annual meeting, according to circumstances. From these societies brief reports of anything important should be sent up to the Medical Journals for the general good. The mingling of the members of the profession at these meetings would not only prove mutually instructive, but highly promotive of social feeling and brotherhood. As doctors, the community cares but little for us except when they are sick. In fact, there is a strong prejudice against the physician. His heavy responsibilities, his irregular life, his anxieties, cares and trials are not appreciated, nor are his labors and charities to the poor and indigent sick, else he would not be subjected to a large specific tax, as is now the case everywhere. Let the members of the profession consider these facts, and stick together. Let there be no petty jealousies harbored toward each other, but let them be friendly and liberal, each guarding with care the feeling and reputation of his medical brethren, and the result will be beneficial, both to the profession and to the public.

